# Reproductive health and endocrine disruption in smallmouth bass (*Micropterus dolomieu*) from the Lake Erie drainage, Pennsylvania, USA

**DOI:** 10.1007/s10661-021-09654-2

**Published:** 2021-12-04

**Authors:** Heather L. Walsh, Sean D. Rafferty, Stephanie E. Gordon, Vicki S. Blazer

**Affiliations:** 1 U.S. Geological Survey, Eastern Ecological Science Center - Leetown Research Laboratory, 11649 Leetown Road, Kearneysville, WV 25430 USA; 2 Pennsylvania Sea Grant College Program, The Pennsylvania State University, Tom Ridge Environmental Center, 301 Peninsula Drive, Erie, PA 16505 USA

**Keywords:** Biological indicators, Reproductive biomarkers, Testicular oocytes, Vitellogenin, Transcript abundance analysis, Indicator species

## Abstract

Smallmouth bass *Micropterus dolomieu* were sampled from three sites within the Lake Erie drainage (Elk Creek, Twentymile Creek, and Misery Bay, an embayment in Presque Isle Bay). Plasma, tissues for histopathological analyses, and liver and testes preserved in RNALater® were sampled from 30 smallmouth bass (of both sexes) at each site. Liver and testes samples were analyzed for transcript abundance with Nanostring nCounter® technology. Evidence of estrogenic endocrine disruption was assessed by the presence and severity of intersex (testicular oocytes; TO) and concentrations of plasma vitellogenin in male fish. Abundance of 17 liver transcripts associated with reproductive function, endocrine activity, and contaminant detoxification pathways and 40 testes transcripts associated with male and female reproductive function, germ cell development, and steroid biosynthesis were also measured. Males with a high rate of TO (87–100%) and plasma vitellogenin were noted at all sites; however, TO severity was greatest at the site with the highest agricultural land cover. Numerous transcripts were differentially regulated among the sites and patterns of transcript abundance were used to better understand potential risk factors for estrogenic endocrine disruption. The results of this study suggest endocrine disruption is prevalent in this region and further research would benefit to identify the types of contaminants that may be associated with the observed biological effects.

**Supplementary information:**

The online version contains supplementary material available at 10.1007/s10661-021-09654-2.

## Introduction

Smallmouth bass (SMB) *Micropterus dolomieu* are apex predators and important sportfish throughout both native and introduced ranges (Noble, 2002). They are native to the Great Lakes region, including the Lake Erie drainage, where angling is economically important and brings in roughly $40.6 million in revenue annually, providing more than $13 million in income for local residents (Graefe et al., 2018). SMB are one of the top four sought after species by anglers in this area (Graefe et al., 2018), which makes them a priority for conservation efforts. In 1987, Presque Isle Bay, located in the Lake Erie drainage in the state of Pennsylvania, was listed as an Area of Concern (AOC; an area that has severe environmental degradation) due to contamination and fish exhibiting a high rate of tumors and other deformities, one of the beneficial use impairments (US EPA, 2020). Sources of contamination included industrial and domestic wastewater, which led to increases in nutrients, heavy metals, and polycyclic aromatic hydrocarbons (PAHs). In 2013, the Bay was delisted (US EPA, 2020), but not without controversy; therefore, there is continued interest in the effects of both legacy and emerging contaminants in these fish populations.

The nearby Susquehanna River watershed in Pennsylvania supports an economically important, although introduced, SMB population. Health issues, including mortality events of young-of-year (Smith et al., 2015; Walsh et al., 2018), as well as skin lesions (Blazer et al., 2020), a high prevalence of intersex (testicular oocytes; TO), and other signs of exposure to endocrine disrupting contaminants in adults (Blazer et al., 2014a) have been observed there. These have not only raised concerns of resource managers and the public but have also been associated with population declines (Schall et al., 2018a; Smith et al., 2015). To date, no single cause for the health anomalies has been identified; rather, poor water quality, complex mixtures of contaminants, and various pathogens and parasites have been identified as potential risk factors (Boonthai et al., 2018; Schall et al., 2018b; Walsh et al., 2018).

Similar population declines of SMB have not been reported in the Great Lakes; however, contaminant exposure and bioaccumulation (Choy et al., 2017; Wallace & Blersch, 2015) have been. SMB and largemouth bass (*Micropterus salmoides*) males sampled at other AOCs in the Great Lakes region have exhibited biomarkers of exposure to estrogenic endocrine disrupting chemicals (EEDCs) including vitellogenin induction and a low to moderate prevalence and severity of TO (Blazer et al., 2018). Although it is unknown if exposure to EEDCs alone in SMB lead to population-level effects, models have suggested a combination of climatic factors and bioactive chemical exposure could have significant population effects (Li et al., 2020). Studies like these have captured the public’s attention not only because of potential effects on sportfishing but also because many of these watersheds are a source of public drinking water. The human population in the Lake Erie drainage is approximately 12 million (US EPA, 2020), and its rivers provide drinking water to roughly 10.6 million of its residents (Myers et al., 2000).

In the current study, biological effects-based monitoring (Ekman et al., 2013) was used to investigate potential reproductive endocrine disruption within Presque Isle Bay and nearby tributaries to Lake Erie within Pennsylvania. Reproductive biomarkers such as TO prevalence and severity, plasma vitellogenin (Vtg), the gonadosomatic index (GSI), and the hepatosomatic index (HSI) were analyzed. Additionally, a suite of gene transcripts in liver and testes associated with reproductive health was developed and transcript abundance measured to provide additional information on associated mechanisms. The results of this study will provide management agencies with information on health conditions that could potentially affect SMB populations in this region.

## Methods

### Landscape analysis

**Fig. 1:**
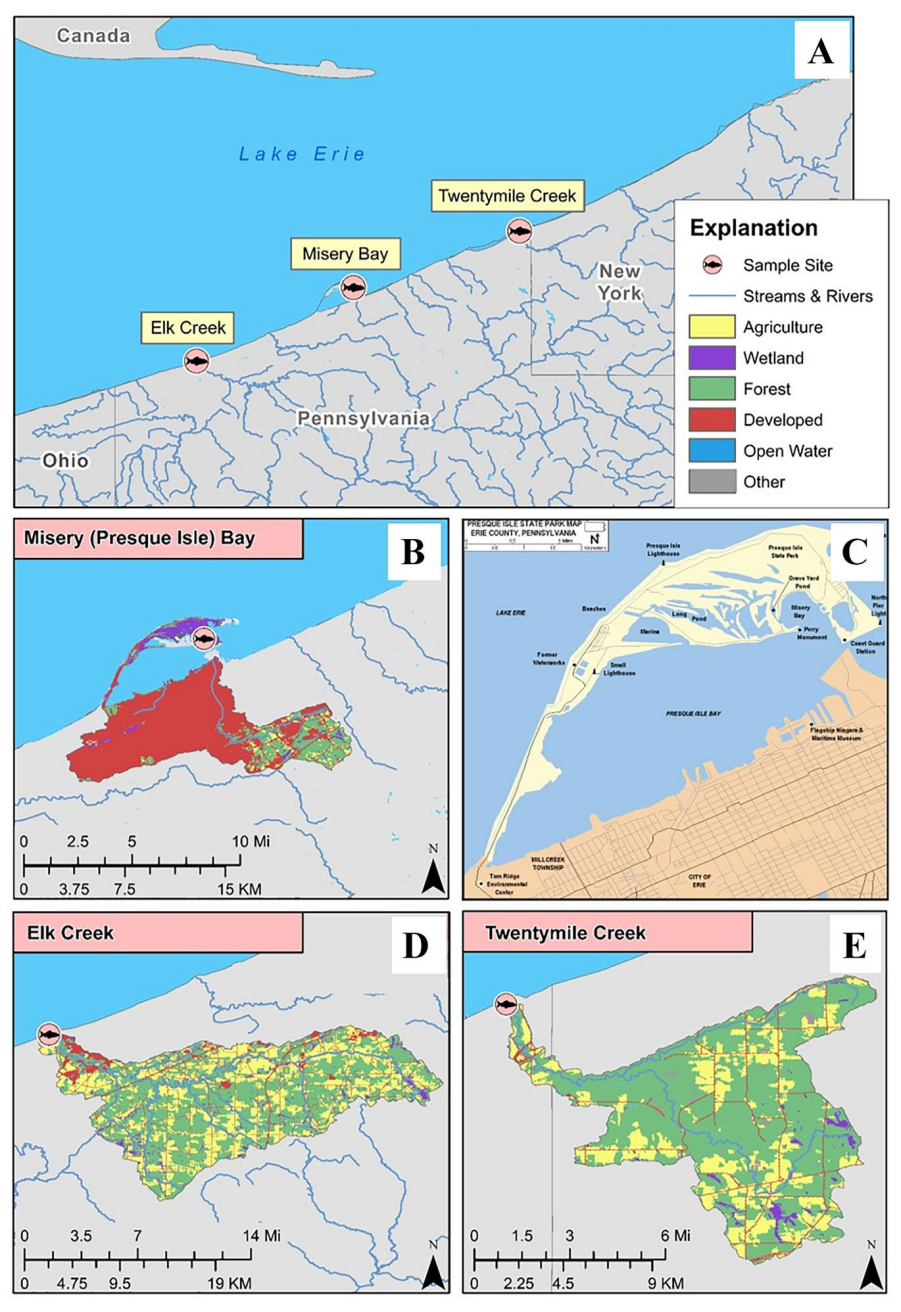
**A** Sampling sites, including Elk Creek, Misery Bay (an embayment in Presque Isle Bay), and Twentymile Creek where smallmouth bass *Micropterus dolomieu* were sampled to assess biomarkers of endocrine disruption. Landuse in the upstream catchments of Misery Bay **B**, **C**, Elk Creek **D**, and Twentymile Creek **E**

On May 9–11, 2016, adult SMB (prespawn) were collected at three sites (Fig. 1) in Erie County, Pennsylvania (PA). These included two Lake Erie tributary sites, Elk Creek (ELK; 42.02464°N, 80.37082°W) and Twentymile Creek (TMC; 42.26113°N, 79.78286°W) and Misery Bay (MB), an embayment within Presque Isle Bay (42.15845°N, 80.08545°W). Landcover data for 2016 was downloaded from https://www.mrlc.gov/data. Stream reach catchments were downloaded as part of the NHDPlusV2 (NHDPlus) geodatabase from http://www.horizon-systems.com/NHDPlus/ NHDPlusV2_data.php and upstream catchments were generated by manually selecting NHDPlus catchments upstream of each site along NHD flowlines. Summaries were generated using Zonal Histogram tool in ArcMap (version 10.6) and are presented as percent landcover using (# of cells per class per catchment) / (total # of cells per catchment) × 100. These summaries included percent water, development, forests, agriculture (pasture/hay and cultivated crops), and wetlands.

### Fish collection

Fish were collected by pulsed-DC boat electrofishing and live fish were transported to an aerated holding tank on site until necropsied (generally within 1 h). Thirty adult (i.e., sexually mature) SMB (> 200 mm) were collected from each location and euthanized with tricaine methane-sulfonate (350 mg/L; Western Chemical Incorporated, Ferndale, Washington) following procedures approved by the Eastern Ecological Science Center and Penn State University Animal Care and Use Committee (IACUC #47,053). Total length (mm) and weight (g) of each fish were measured to the nearest unit in the field; external abnormalities were documented; and fish were bled with heparinized syringes from the caudal vein. Blood was placed into heparinized vacutainer tubes and held on wet ice until returned to the laboratory (within 4 h) at which time samples were centrifuged at 3000 rpm for 10 min. Plasma was aseptically aliquoted into cryovials and stored at – 80 ℃.

Fish were dissected and the gonads and liver were excised and weighed (g). The gonadosomatic index was calculated as follows: (gonad weight / body weight) × 100 and the hepatosomatic index was calculated as follows: (liver weight / body weight) × 100. Small pieces of liver were placed in separate cryovials with Invitrogen RNAlater® Stabilization Solution (ThermoFisher, Waltham, Massachusetts) for RNA preservation. Additionally, from a subset of males from each site (approximately 10) a piece of testis was preserved in RNAlater®. For each fish, one whole gonad (or multiple pieces if large) and 5–6 pieces of liver were placed in Z-Fix® (Anatech Ltd., Battle Creek, Michigan) preservative. Finally, sagittal otoliths were removed for age analysis as previously described in Blazer et al. (2014b).

### Histology

Multiple (five to eight) cross-sections of the gonad and at least three cross-sections of the liver were placed in cassettes for histological processing, embedded into paraffin, sectioned at 6 µm, and routinely stained with hematoxylin and eosin (Luna, 1992). Gonadal maturation stage was assessed as previously described in Blazer (2002). TO severity rankings ranging from 0 (not present) to 4 (severe) previously described in Blazer et al. (2007) were used for each cross-section. A mean severity from a minimum of five cross-sections (along the length of the testes) was obtained and based on that mean individuals were ranked low (0–0.5), medium (0.6–1.5), and high (> 1.6) severity.

### Plasma vitellogenin quantification

Plasma Vtg concentrations were measured using a direct enzyme-linked immunosorbent assay (ELISA) with monoclonal antibody 3G2 (Caymen Chemical, Ann Arbor, Michigan) as previously described (Blazer et al., 2014b; Denslow et al., 1999). Briefly, plasma samples were diluted as necessary in PBSZ-AP (10 mM phosphate, 150 mM NaCl, 0.02% azide, pH 7.6). SMB Vtg was used as a standard for all plasma analyzed. The Vtg standards were prepared at the University of Florida, Department of Physiological Sciences from plasma of 17β-estradiol exposed male SMB held at the U.S. Geological Survey Eastern Ecological Science Center at the Leetown Research Laboratory, Kearneysville, West Virginia. Optical density was measured on a multiwell plate reader (SpectraMax M4, Molecular Devices Inc., Sunnyvale, California) at 405 nm. Concentrations of the unknowns were determined from the standard curves with the Softmax Pro TM Program version 7.1.0 (Molecular Devices Inc., San Jose, California). Limit of detection was 1 µg/mL and inter- and intra-assay variability was < 10%.

### RNA extractions

Total RNA was extracted from 10 to 25 mg of tissue with an E.Z.N.A.® Total RNA Kit (Omega Bio-Tek, Norcross, Georgia) following manufacturer’s protocols and stored at – 80 °C until use. All samples were quantified with a Qubit 3 Fluorometer with an RNA HS Assay Kit (Agilent, Santa Clara, California).

### Testes transcriptome assembly

The testes transcriptome was previously developed from SMB sampled in the Chesapeake Bay watershed. Total RNA from 13 males without TO, 10 males with TO, and 15 immature females was pooled separately (to create three pooled samples), which were used as template for RNA-Seq libraries. The pooled sample of non-intersex males consisted of total RNA from the testes of seven males from experimental ponds at the Leetown Research Laboratory, two males from Bald Eagle Creek, and four males from Wyalusing Creek (tributaries of the Susquehanna River in Pennsylvania). The pooled sample of immature females consisted of total RNA from the ovary of 15 SMB sampled from Shultz’s Fish Hatchery in Lake Ariel, Pennsylvania. The pooled sample of intersex males consisted of total RNA from two males from the Juniata River, one male from Chillisquaque Creek (both tributaries of the Susquehanna River in Pennsylvania), and seven males from the Shenandoah River in Virginia (a tributary of the Potomac River). Prior to pooling these samples, each individual sample was normalized to the sample with the lowest concentration of RNA. After pooling, samples were analyzed with an Agilent RNA 6000 Nano Kit on an Agilent 2100 Bioanalyzer (Agilent, Santa Clara, California). Three Illumina TruSeq Stranded mRNA, 150-base pair (bp), paired-end libraries with poly(A) selection were constructed at the Institute for Genome Sciences (IGS) in Baltimore, Maryland, and sequenced on an Illumina HiSeq 4000 (Illumina, Inc., San Diego, California). The reads from these libraries were used for the de novo transcriptome assembly (Bioproject Accession No. PRJNA474933).

Reads were trimmed of adapters by IGS and analyzed for quality with FastQC (Andrews, 2010). A total of 108,544,928, 94,265,096, and 92,702,334 150 bp reads were obtained with RNA-Seq. Based on FastQC results, the first 10 and last 15 bases were trimmed from all reads to improve sequence quality. The assembly program Trinity was used to create a de novo transcriptome assembly (Bolger et al., 2014). Parameters were set for stranded, paired-end reads (–SS_lib_type RF) and trimming parameters for the Trimmomatic (Haas et al., 2013) plugin were set to crop the first 15 and last 10 bases of all reads. Trimmed reads were mapped back to the assembly with Bowtie2 (Langmead & Salzberg, 2012) to analyze assembly quality. The CD-HIT program (Limin et al., 2012; Weizhong & Godzik, 2006) was used with the CD-HIT-EST package with default settings to cluster similar transcripts and produced a 96.5% overall alignment rate. Next, overlap and redundancy and multiple small isoforms were collapsed into one long isoform and sequences that did not contain an open reading frame were removed with EvidentialGene (Gilbert, 2013) with default settings. After elimination of similar transcripts with CD-HIT and removal of redundant isoforms with EvidentialGene, the Trinity assembly consisted of a total of 50,892 transcripts with an average length of 1359.68 bp (Table S1). Assembly statistics were analyzed with Transrate (Smith-Unna et al., 2016) with default settings. Finally, transcripts were annotated with the program Diamond (Butchfink et al., 2015) with a database consisting of four fish species: zebrafish *Danio rerio*, barramundi *Lates calcarifer*, medaka *Oryzias latipes*, and rainbow trout *Oncorhynchus mykiss*. The sensitive mode was chosen in Diamond to accommodate longer sequences.

### Nanostring CodeSet development

Nanostring nCounter® (Nanostring Technologies, Inc., Seattle, Washington) technology, a direct digital multiplex method, was used to quantify transcripts potentially associated with reproductive health. The testes CodeSet, including 43 transcripts (Table S2), was developed from the transcriptome analyses described above. The transcripts were previously identified as biomarkers of reproduction or associated with TO or endocrine disruption in other fish species (Baron et al., 2005; Deloffre et al., 2012; Depiereux et al., 2014; Garcia-Reyero et al., 2009; Hahn et al., 2016; Kishi et al., 2006; Zhao & Hu, 2012). Twenty-one liver transcripts (Table S3) from a previous study (Hahn et al., 2016) were also used. CodeSet development consisted of analyzing sequences identified from the transcriptome in Geneious 10.1.3 (https://www.geneious.com) to detect coding regions and annotation was confirmed with NCBI Blastx (Kulkarni, 2011). The Nanostring nCounter® analysis was carried out at the Leetown Research Laboratory according to manufacturer’s protocols with 50 ng of purified RNA. Transcripts were normalized in nSolver 4.0 with positive controls (spike-in oligos), negative controls, and housekeeping transcripts (Tables S2, S3) included in the Nanostring CodeSet. The limit of detection was determined as the mean of the negative controls + 2 × the standard deviation and was 32 for liver transcripts and 27 for testes transcripts.

### Statistical analyses

All statistical methods were conducted in R × 64 4.0.0 (R Core Team, 2020). A Kruskal–Wallis one-way ANOVA followed by a Dunn’s multiple comparison test (package “dunn.test”) with a Bonferroni correction was conducted to identify differences in biological variables (GSI, HSI, TO and plasma Vtg) and testes and liver transcript abundance among sites. A Kruskal–Wallis test was also conducted to identify differences in  liver transcript abundance between males and females. Liver transcripts that were not significantly different between sexes were analyzed together and transcripts that were significantly different were analyzed separately by sex. A Fisher’s exact test was used to identify significant differences in the prevalence of TO. Finally, a Spearman’s rank correlation analysis (function “rcorr”) was conducted to identify significant associations between biological variables and liver and testes transcripts. Results with a *p* value < 0.05 were considered statistically significant. Differential expression analysis of liver transcripts from males with low, medium, or high TO ranks compared to females was performed with NanoStringDiff (Wang et al., 2016). For this analysis, original count data was used instead of the normalized data from nSolver 4.0 since NanoStringDiff performs its own normalization. A false discovery rate (FDR) < 0.05 and log fold-change of >  ± 1.5 was considered statistically significant since fold-change values < 1.5 could be considered background noise (Dalman et al., 2012; Zhao et al., 2018).

## Results

### Land use

Drainage size and percent landcover varied among sites (Table 1). ELK had the largest upstream drainage (255.8 km^2^) and the greatest amount of agricultural landcover (33.4%), primarily in the form of pasture (20.5%) versus cultivated crops (12.9%). MB had the smallest drainage size (76.6 km^2^), the greatest amount of developed landcover (72%) and the least amount of agricultural landcover (3.1%) which was similarly dominated by pasture (2.9%). The TMC drainage had the greatest amount of forested landcover (67.6%) and ranked third in amount of development (4.3%), but second in agriculture (24.3%).

**Table 1:** Landcover summaries for smallmouth bass sampling sites in the Lake Erie drainage

	**Misery Bay (MB)**	**Elk Creek (ELK)**	**Twentymile Creek (TMC)**
Watershed area (sq km)	76.6	255.8	90.2
Water (%)	1.6	0.2	0.2
Developed (%)	72.0	9.0	4.3
Forest (%)	15.4	53.0	67.6
Agriculture (%)	3.1	33.4	24.3
Pasture/Hay (%)	2.9	20.5	21.5
Cultivated crops (%)	0.2	12.9	2.8
Wetlands (%)	6.5	3.6	2.1
Other (%)	1.4	0.8	1.5

### Fish morphometrics

Available data can be found in Walsh et al. (2021). There were 26 (26/30, 87%), 11 (11/30, 37%), and 16 (16/30, 53%) males collected at MB, ELK, and TMC, respectively. There was no significant difference among the sites in length, age, or HSI of male SMB, although those from ELK weighed less than those at the other two sites (Table 2).

**Table 2:** Morphometric and reproductive indicators of male and female smallmouth bass captured at study sites

	**Misery Bay (MB)**	**Elk Creek (ELK)**	**Twentymile Creek (TMC)**
***Male***^***a***^	***n*** = ***26***	***n*** = ***11***	***n*** = ***16***
Length (mm)	413.9 ± 7.3^**a**^	374.4 ± 17.4^**a**^	404.9 ± 9.5^**a**^
Weight (gm)	1239.5 ± 60.0^**a**^	836.5 ± 118.0^**b**^	1027.5 ± 71.0^**a**^
Age	6.5 ± 0.3^**a**^	5.2 ± 0.5^**a**^	7.1 ± 0.7^**a**^
Hepatosomatic Index (HSI)	1.9 ± 0.1^**a**^	1.5 ± 0.2^**a**^	2.0 ± 0.1^**a**^
Gonadosomatic Index (GSI)	1.1 ± 0.1^**a**^	1.0 ± 0.1^**a**^	1.2 ± 0.1^**a**^
Plasma Vitellogenin (µg/ml)	17.0 ± 1.6^**a**^	21.0 ± 4.5^**a**^	66.4 ± 11.6^**b**^
Testicular Oocyte Severity	1.2 ± 0.2^**ab**^	1.5 ± 0.1^**a**^	0.8 ± 0.1^**b**^
Testicular Oocytes (%)	96^**a**^	100^**a**^	88^**a**^
***Female***	***n*** = ***4***	***n*** = ***19***	***n*** = ***14***
Length (mm)	424.3 ± 18.7^**a**^	405.6 ± 9.1^**a**^	401.3 ± 15.2^**a**^
Weight (gm)	1298.5 ± 188.0^**a**^	1101.1 ± 88.7^**a**^	1044.5 ± 110.2^**a**^
Age	7.8 ± 1.0^**a**^	6.5 ± 0.7^**a**^	7.2 ± 1.0^**a**^
Hepatosomatic Index (HSI)	2.4 ± 0.2^**a**^	2.1 ± 0.2^**a**^	2.7 ± 0.2^**a**^
Gonadosomatic Index	9.9 ± 1.7^**a**^	9.5 ± 0.7^**a**^	10.4 ± 1.0^**a**^
Plasma vitellogenin (µg/ml)	308.8 ± 30.4^**a**^	330.2 ± 18.2^**a**^	636.7 ± 40.6^**b**^

^a^Values (means ± standard error) followed by the same lowercase letters were not significantly different (*p* > 0.05)

At ELK there were more females collected than males, while at the other two sites more males were collected. There were no significant differences in length, weight, age, or HSI among sites for female SMB (Table 2).

### Reproductive biomarkers

**Fig. 2:**
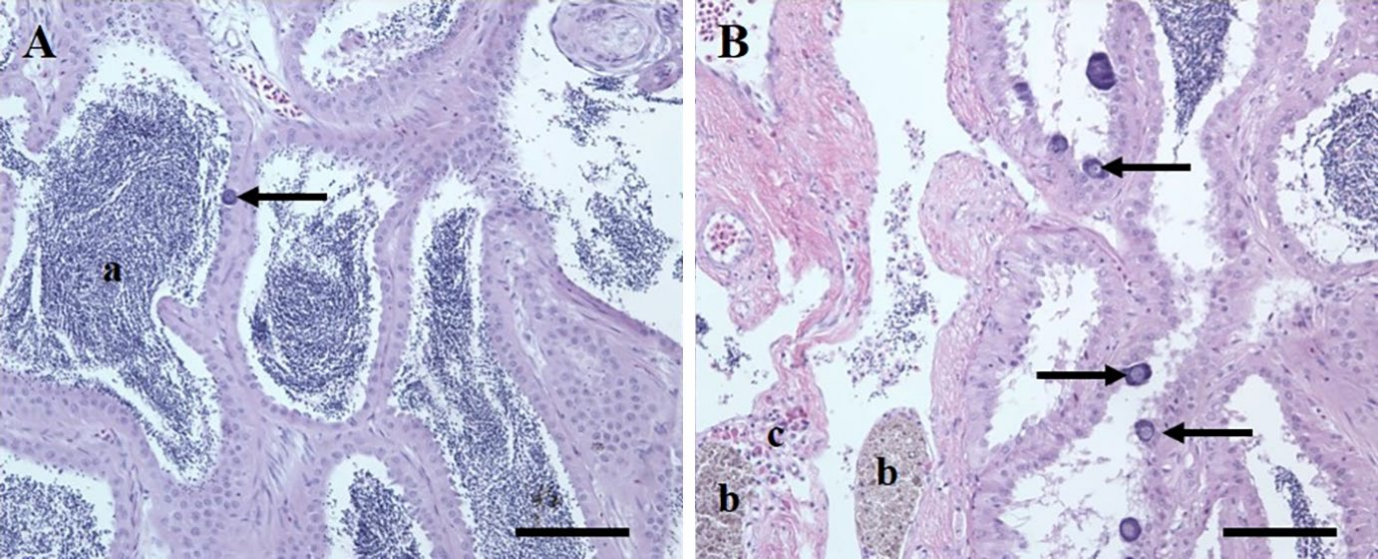
Primary oocytes in testicular tissue of smallmouth bass. **A** Low severity testicular oocytes. One oocyte (arrow) observed in the section with sperm (a) present. H & E stain. Scale bar equals 100 µm. **B** Moderate severity testicular oocytes with multiple oocytes (arrows). Focal accumulations of ceroid/lipofuscin or testicular macrophageaggregates (b) and eosinophils (c). H&E stain. Scale bar equals 100 µm

Histology revealed that TO did not advance past the primary oocyte (Fig. 2) and that all fish (males and females) were at the same reproductive stage (prespawn). At all sites TO prevalence was high, ranging from 100% at ELK to 88% at TMC, and there was no significant difference among sites. Mean TO severity was significantly lower at TMC when compared to ELK, while MB was intermediate (Table 2). Male GSI was not different among the sites and was positively correlated with age (*p* = 0.001, rho = 0.45) and HSI (*p* = 0.037, rho = 0.29). Plasma Vtg was significantly higher in male SMB at TMC than the other sites, which were not different (Table 2), and was positively correlated with GSI (*p* = 0.003, rho = 0.49) and age (*p* = 0.006, rho = 0.47) and negatively correlated with intersex severity (*p* = 0.036, rho =  − 0.23). There was no significant difference in GSI in female SMB among sites, and when sites were combined GSI was positively correlated with age (*p* = 0.0.015; rho = 0.40) and HSI (*p* = 0.014, rho = 0.41). As in males, plasma Vtg was significantly higher at TMC than at the other sites (Table 2) and was not significantly correlated with any other biological variables.

### Liver transcript abundance

Transcript abundance of four housekeeping and 17 genes associated with reproductive function, endocrine activity, and contaminant detoxification pathways was measured (Table 3). Six of these transcripts (*ahr*, *cyp1a*, *cyp3a*, *erβ1*, *mt*, and *thrβ*) were not significantly different between males and females when they were pooled across sites. Hence, males and females were combined to evaluate site differences. Only one transcript, *erβ1*, was significantly higher at TMC than ELK (*p* < 0.001) and MB (*p* = 0.013).

**Table 3:** Liver transcripts included in the Nanostring nCounter® analyses of transcript abundance

**Transcript name**	**Transcript symbol**
40S ribosomal protein S12*	40SrpS12*
Elongation factor 1A*	ef1α*
Eukaryotic translation initiation factor 3D*	etif3d*
Ribosomal protein L8*	rpl8*
17-beta hydroxysteroid dehydrogenase	*17βhd*
Androgen receptor alpha	*arα*
Androgen receptor beta	*arβ*
Aryl hydrocarbon receptor	*ahr*
Choriogenin	*chg*
CYP1A	*cyp1*α
CYP3A	*cyp3*α
Estrogen receptor A	*erα*
Estrogen receptor beta 1	*erβ1*
Estrogen receptors beta 2	*erβ2*
Glucokinase	*glk*
Insulin-like growth factor 1	*igf1*
Metallothionein	*mt*
Thyroid hormone receptor beta	*thrβ*
Type I deiodinase	*dio1*
Type II deiodinase	*dio2*
Vitellogenin	*vtg*

^*^Indicates housekeeping transcripts

**Fig. 3:**
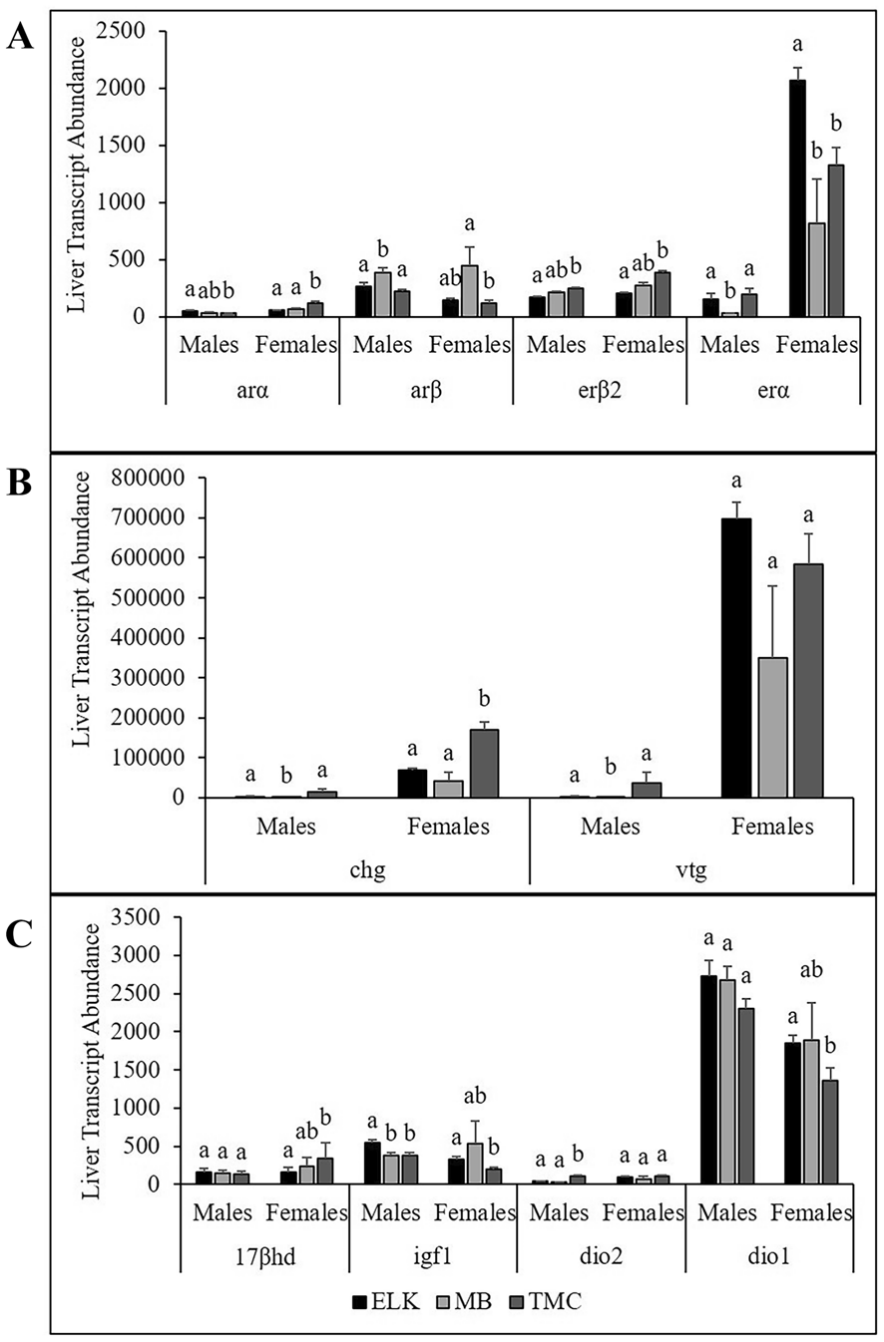
**A** Liver transcript abundance of reproductive transcripts including androgen receptor alpha (*arα*), androgen receptor beta (*arβ*), estrogen receptor beta (*erβ2*), and estrogen receptor alpha (*erα*); **B** choriogenin (*chg*), and vitellogenin (*vtg*); and **C** 17-beta hydroxysteroid dehydrogenase (*17βhd*), insulin-like growth factor 1 (*igf1*), iodothyronine deiodinase 2 (*dio2*), iodothyronine deiodinase 1 (*dio1*). Bars with the same lowercase letters indicate no significant difference (*p* > 0.05)

There were 11 liver transcripts (*arα*, *arβ*, *erβ2*, *erα*, *17βhd*, *glk*, *igf1*, *dio2*, *dio1*, *chg*, and *vtg*) that were significantly different between males and females. The trend among sites for abundance of *arα* was opposite in males and females (Fig. 3A). Males from ELK had the highest, TMC the lowest, and MB intermediate, while females at TMC had the highest transcript abundance and ELK the lowest. Abundance of *arβ* showed a similar trend in males and females with MB having the highest abundance. For estrogen receptors, the highest abundance of *erβ2* was observed at TMC with MB intermediate and ELK lowest in both sexes. Females at ELK had a greater abundance of *erα* than MB and TMC whereas males from ELK and TMC had a greater abundance than MB (Fig. 3A).

The abundance of two transcripts associated with oocyte development, *chg* and *vtg*, were measured. In both males and females, *chg* was highest at TMC*.* Abundance of *vtg* was lowest at MB in both sexes, although not significant in females (Fig. 3B). A third transcript, *17βhd* which is involved in sex steroid regulation, was also detected. In males, there were no significant site differences in *17βhd*, while in females *17βhd* was lowest at ELK and highest at TMC (Fig. 3C).

Abundance of *igf1* was highest in males at ELK and highest in females (although not significantly different) at MB. In transcripts associated with thyroid hormone regulation, *dio2* was low in both sexes and only higher in males at TMC than the other sites. The abundance of *dio1* was significantly greater than *dio2* and there were no site differences in males while in females from TMC *dio1* transcript abundance was less than ELK (Fig. 3C).

### Testes transcript abundance

**Fig. 4:**
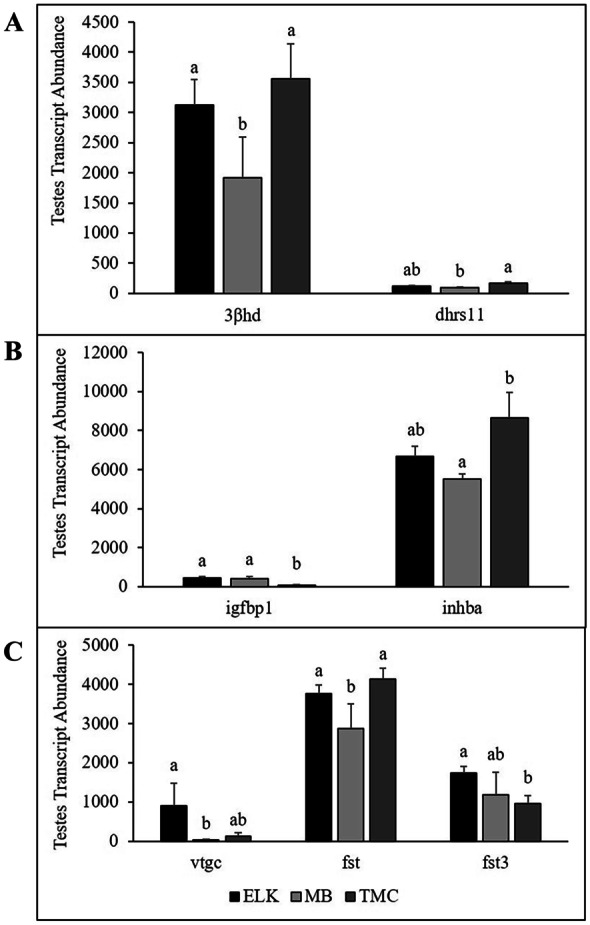
**A** Testes transcript abundance of transcripts associated with steroid biosynthesis including 3-beta hydroxysteroid dehydrogenase (*3βhd*), dehydrogenase/reductase SDR family member 11 (*dhrs11*); **B** insulin-like growth factor binding protein 1 (*igfbp1*), and inhibin subunit beta A (*inhba*), and female reproductive transcripts vitellogenin C (*vtgc*), follistatin (*fst*), and follistatin 3 (*fst3*). Bars with the same lowercase letters indicate no significant difference (*p* > 0.05)

A total of 40 transcripts associated with male and female reproduction and three housekeeping transcripts were quantified in the testes of 10 males from each site (Table 4). Seven transcripts (*3βhd*, *dhrs11*, *igfbp1*, *inhba*, *vtgc*, *fst*, and *fst3*) were significantly different among sites. For transcripts associated with steroid biosynthesis, there was a greater abundance of *3βhd* at TMC and ELK than MB and a greater abundance of *dhrs11* at TMC than MB with ELK intermediate (Fig. 4A). Transcripts of *igfbp1* were greater at ELK and MB than TMC, while *inhba* transcript abundance was greater at TMC than MB, with ELK intermediate (Fig. 4B). Transcripts associated with female reproduction and oocyte development that were significantly different among sites included *vtgc*, *fst*, and *fst3. Vtgc* abundance was greater at ELK than MB with TMC intermediate, while transcripts of *fst3* were greater at ELK than TMC with MB intermediate. *Fst* transcripts were greater at ELK and TMC than MB (Fig. 4C).

**Table 4:** Testes transcripts included in the Nanostring nCounter® analyses of transcript abundance

**Transcript name**	**Transcript symbol**
40S ribosomal protein S18*	40srps18*
Beta-actin*	βactin*
Eukaryotic translation initiation factor 3D*	etif3d*
17-beta hydroxysteroid dehydrogenase	*17βhd*
3-beta hydroxysteroid dehydrogenase	*3βhd*
Aromatase	*cyp19a1a*
Cytochrome P450 11B	*cyp p450 11b*
Dehydrogenase reductase SDR family member 11	*dhrs11*
Doublesex and mab-3 related transcription factor 1	*dmrt1*
Doublesex and mab-3 related transcription factor 2	*dmrt2*
Doublesex and mab-3 related transcription factor 3	*dmrt3*
Follistatin	*fst*
Follistatin 3	*fst3*
Gonadotropin releasing hormone receptor	*grhr*
Homeobox protein NOBOX	*nobox*
Inhibin alpha	*inhα*
Inhibin beta b	*inhβb*
Insulin like growth factor binding protein 1	*igfbp1*
Insulin like growth factor binding protein 2A	*igfbp2a*
Insulin like growth factor binding protein 3	*igfbp3*
Insulin like growth factor binding protein 5	*igfbp5*
Luteinizing hormone receptor	*lhr*
Nanos	*nanos*
Nuclear receptor subfamily 0 group B member 1	*nrs0b1*
P43 5S RNA-binding protein	*42sp43*
Relaxin receptor 2	*rr2*
Sperm flagellar protein 1	*spef1*
Sperm flagellar protein 2	*spef2*
Sperm surface protein SP17	*sp17*
SRY-box transcription factor 7	*sox7*
SRY-box transcription factor 9B	*srybox9b*
Steroidogenic Acute Regulatory Protein	*star*
Synaptonemal complex protein 1	*sycp1*
Uncharacterized protein 1	*up1*
Uncharacterized protein 2	*up2*
Vitellogenin	*vtg*
Vitellogenin C	*vtgc*
Wnt family member 5B	*wnt5b*
Zona pellucida 3	*zp3*
Zona pellucida 3iX1	*zp3ix1*
Zona pellucida 4	*zp4*
Zona pellucida AX	*zpax*
Zygote arrest 1-like	*zar1*

^*^Indicates housekeeping transcripts

### Transcript abundance association with other biological endpoints

The association of transcript abundance with other biological indicators was evaluated by combining data of male SMB from all sites. Few of the testes transcripts were significantly correlated with biological variables (Table 5). GSI was negatively correlated with *fst3*. There were no transcripts correlated with TO prevalence; however, TO severity was positively correlated with *igfbp1*. Plasma Vtg was positively correlated with *3βhd*, *drsdr11*, and *fst3*. Only one liver transcript, *dio1*, was positively associated with TO severity. Male plasma Vtg was positively associated with liver transcript abundance of *chg, erα*, *vtg*, and *cyp3a*, while it was negatively correlated with abundance of *erβ1*, *dio1*, *arα*, and *arβ*. Male GSI was positively correlated with *cyp3a*, *chg*, and *erα*, and negatively correlated with *arβ* and *thrβ* (Table 5).

**Table 5:** Statistically significant correlations between biological variables and transcript abundance in male and female smallmouth bass. Values were considered statistically significant at *p* < 0.05

**Transcript Group**	**Biological variable**	**Transcript**	***p*** value	**Spearman’s rho**
Testes reproductive male bass	Gonadosomatic index	*fst3*	0.020	− 0.42
	Intersex severity	*igfbp1*	0.025	0.40
	Plasma vitellogenin	*3βhd*	0.006	0.50
		*dhrs11*	0.035	0.39
		*fst*	0.003	0.63
Liver male bass	Gonadosomatic index	*arβ*	0.041	− 0.28
		*cyp3a*	0.043	0.28
		*chg*	0.009	0.36
		*erα*	0.012	0.34
		*thrβ*	0.027	− 0.31
	Testicular oocyte severity	*dio1*	0.042	0.28
	Plasma vitellogenin	*arα*	0.012	− 0.35
		*arβ*	0.028	− 0.47
		*ahr*	< 0.001	− 0.48
		*cyp3a*	0.036	0.30
		*erα*	< 0.001	0.67
		*erβ1*	0.022	− 0.32
		*vtg*	< 0.001	0.49
		*chg*	< 0.001	0.56
		*dio1*	0.031	− 0.31
Liver female bass	Gonadosomatic index	*mt*	0.029	− 0.37
		*thrβ*	0.023	− 0.38
		*dio2*	0.042	− 0.34
	Plasma vitellogenin	*17βhd*	< 0.001	0.66
		*arα*	< 0.001	0.68
		*erβ1*	< 0.001	0.65
		*erβ2*	< 0.001	0.69
		*chg*	< 0.001	0.61
		*igf1*	0.012	− 0.41
		*dio1*	< 0.001	− 0.67

In females, abundance of *17βhd*, *arα*, *chg*, *erβ1*, and *erβ2* were positively correlated with plasma Vtg but negatively correlated with *igf1* and *dio1*. Female GSI was negatively correlated with *mt*, *thrβ*, and *doi2* (Table 5).

Differential expression analysis of liver transcripts in males by TO ranking (low, medium, high) versus immature females was conducted to better assess potential feminization (Table 6). Transcripts *chg*, *era*, and *vtg* were significantly down regulated in all male groups when compared to female; however, the log-fold change when compared to medium TO severity males was less than compared to low TO severity males. The same decrease was not seen in high TO males, which may have been an artifact of the low numbers of high TO males in the dataset. Interestingly, *ara* and *glk* had decreasing log-fold changes among low, medium, and high TO severity ranks. There was no difference in *erβ1* or *erβ2* between females and any group of males.

**Table 6:** Log fold-change (logFC), likelihood-ratio (lr), and false discovery rate (FDR) of liver transcripts from male smallmouth bass with low, medium, and high testicular oocyte (TO) severity compared to females. Transcripts were significantly differentially expressed with a FDR < 0.05 and logFC >  ± 1.5 and are highlighted in gray

**Transcript**	**Low TO severity males vs. females**			**Med TO severity males vs. females**			**High TO severity males vs. females**		
	**logFC**	**lr**	**FDR**	**logFC**	**Lr**	**FDR**	**logFC**	**lr**	**FDR**
*ahr*	0.2	0.338	0.609	0.0	0.002	0.968	0.5	3.834	0.074
*arα*	− 1.6	18.827	0.001	− 1.2	17.649	0.000	− 1.1	10.955	0.002
*arβ*	1.1	14.303	0.001	0.9	11.663	0.001	1.1	15.929	0.000
*chg*	− 5.0	26.839	0.001	− 3.7	27.525	0.000	− 6.2	46.408	0.000
*cyp1a*	0.2	0.954	0.411	− 0.1	0.357	0.629	0.4	5.802	0.032
*cyp3a*	− 0.1	0.334	0.609	− 0.2	2.252	0.198	0.0	0.034	0.853
*dio1*	0.5	8.129	0.010	0.5	12.065	0.001	0.8	25.632	0.000
*dio2*	− 0.7	3.570	0.102	− 0.5	2.672	0.157	− 1.3	15.210	0.000
*erα*	− 4.5	48.351	0.000	− 4.0	65.466	0.000	− 4.4	59.331	0.000
*erβ1*	− 0.4	1.448	0.327	0.1	0.072	0.809	− 0.3	0.989	0.400
*erβ2*	− 0.4	4.077	0.087	− 0.5	9.280	0.004	− 0.4	5.331	0.036
*glk*	− 2.8	19.311	0.000	− 1.9	15.980	0.000	− 1.2	5.489	0.036
*igf1*	0.5	3.850	0.092	0.3	1.931	0.235	0.6	5.447	0.036
*mt*	− 0.5	2.150	0.228	− 0.3	1.446	0.296	0.1	0.246	0.670
*thrβ*	0.0	0.069	0.814	− 0.1	0.523	0.587	0.2	1.463	0.292
*vtg*	− 9.5	37.921	0.000	− 4.5	23.027	0.000	− 9.0	45.920	0.000
*17βhd*	− 0.9	17.667	0.000	− 0.7	16.067	0.000	− 0.7	12.631	0.001

## Discussion

This study determined that commonly measured signs of estrogenic exposure such as intersex (TO) and plasma Vtg were present in male SMB captured at all three sites within the Lake Erie drainage with varying landcover/land-use. The highest (72.0%) developed land use was around MB, with the other two sites much lower (9.0% at ELK and 4.3% at TMC). ELK had substantially more agricultural land than the other two sites while TMC drainage area had roughly 15% and 52% more forest cover than ELK and MB, respectively. Despite these differences, SMB from all three sites demonstrated a high rate (88–100%) of TO. ELK, with the highest agricultural land use, had the highest prevalence and severity of TO. Conversely, both male and female bass from TMC, the site with the highest percent of pasture/hay (21.5%) and forested landcover, had the highest plasma Vtg concentrations and the highest *vtg* transcript abundance in males. In previous studies, TO in bass have been associated with agricultural land use and pesticide/herbicide exposure (Abdel-Moneim et al., 2017; Bizarro et al., 2014; Kolpin et al., 2013), while other studies have shown an association with polycyclic aromatic hydrocarbons (PAHs), polychlorinated biphenyls (PCBs), and industrial EDCs (LaPlaca, 2017; Lee Pow et al., 2017), more typically associated with developed land use.

It is generally thought that TO are induced early in life during sexual differentiation (Krisfalusi & Nagler, 2000; Metcalfe et al., 2010), although severity can increase over time (Lange et al., 2009). Conversely, plasma Vtg and liver *vtg* transcripts are likely indicative of a more recent exposure to EEDCs (Hemmer et al., 2002; Korte et al., 2000). As in other studies, these changes observed in adult SMB are likely induced by exposure to complex mixtures throughout their life. Given the higher plasma Vtg concentrations in both males and females from TMC, the site with the most forested landcover, it would be beneficial to evaluate not only anthropogenic chemicals but also natural hormones from human wastewater and animal feeding operations that can reach the aquatic environment by practices such as biosolid application or other nutrient enrichment practices (Ciparis et al., 2012; Liu et al., 2009). Phytoestrogens, estrogen-like compounds naturally occurring in plants, are compounds that can also increase in the aquatic environment through these practices and other sources (Burnison et al., 2003; Hoerger et al., 2009; Kolpin et al., 2010). Exposure to phytoestrogens has been associated with intersex in wild fishes. Wang et al. (2020) demonstrated that the agonistic activity of equol to *erα* in wild mullet *Mugil soiuy* was 3.5 times higher than that of medaka, correlating to a stronger potential to induce intersex. In sturgeon fed the phytoestrogen daidzein, *erα* and *erβ* were significantly upregulated in both normal and intersex males when compared to controls, while females, normal, and intersex males all had upregulated *vtg* (Fajkowska et al., 2021).

It has been suggested that knowledge of transcriptional changes at the organismal level can be used by scientists and management agencies as early warning signals of potential population-level effects (Connon et al., 2012), which could be used to support managerial action. However, numerous studies have measured gene expression in wild fishes associated with intersex and vitellogenin induction in male fishes with varying results (Abdel-Moneim et al., 2017; Bahamonde et al., 2015; Sardi et al., 2015). This is likely due to species, seasonal and reproductive stage differences, as well as differences in the complex mixtures of chemicals and other stressors wild fishes are exposed to. The results of a modeling study in English rivers concluded that widespread feminization (TO, plasma Vtg, feminized reproductive duct) was multifactorial and included steroidal estrogens, xenoestrogens, and antiandrogens (Jobling et al., 2009). In this study, we used transcript abundance to better understand the risk factors associated with reproductive endocrine disruption, looking for associations among the sites and associations among transcript abundance and other biological responses. Caveats to consider include that transcription occurs as a response to a changing environment, both internally and externally, and is an attempt of the organism to respond to those changes. Additionally, sportfish and apex predators such as SMB tend to move, sometimes long distances, and often in and out of the tributaries they use for spawning (Schall et al., 2019). It is likely the SMB collected in this study spent at least part of their life in the lake and transcriptional changes may reflect those environmental differences.

Since transcript abundance is most indicative of the current exposures or conditions, the lack of association with TO prevalence is not surprising. Plasma Vtg also did not correlate with TO prevalence or severity, a finding similar to reports from other species (Bizarro et al., 2014; Kirby et al., 2004). TO severity was positively correlated with *igfbp1*, insulin-like growth factor binding protein 1, in the testes and hepatic *dio1.* Fish possess a suite of igfbps that influence the activities of the insulin and insulin-like growth factor systems (Allard & Duan, 2018; Duan & Xu, 2005). In rainbow darter *Etheostoma caeruleum*, *igfbp1* was associated with ovulation in response to estrogen exposure; however, it was downregulated in intersex males (Bahamonde et al., 2015) and was not detected in preovulatory follicles in rainbow trout *Oncorhynchus mykiss* (Kamangar et al., 2006). Conversely, tilapia *Oreochromis niloticus* exposed as sac fry to 17β-estradiol and 4-nonylphenol for 21 days, then held for 112 days, showed significantly increased hepatic *igfbp1* (Celino-Brady et al., 2019). The association of TO severity with insulin-related regulatory factors is interesting given the finding that metformin, a commonly prescribed diabetic medication, induces intersex (Niemuth & Klaper, 2015) and *igfbp1* production is regulated by insulin (Brismar et al., 1994). Further research is required to understand these interactions.

The other transcript, *dio1* (deiodinase type 1), that was positively associated with TO severity was negatively correlated with plasma Vtg in both male and female bass. Conversely, *dio2* and *thrβ* (thyroid receptor β) were negatively correlated with female GSI, while only *thrβ* was negatively associated with male GSI. The thyroid plays a role in regulating the HPG (hypothalmus-pituitary-gonad) axis and hence reproductive physiology. However, this regulation may be dependent on species, sex, and reproductive stage (Deal & Volkoff, 2020). Both type 1 and type 2 deiodinases, *dio1* and *dio2*, are important in controlling thyroid activity by activating T4 to T3 conversion but their expression and activity may differ depending on the species, tissue, and stimuli (Jarque & Pina, 2014). Hepatic *dio2* transcripts were preferentially expressed in the liver of rainbow trout *Oncorhynchus mykiss* (Sambroni et al., 2001) and were more abundant in liver of walleye *Sander vitreus* (Picard-Aitken et al., 2007) than *dio1*, which is opposite of observations in this study. However, in zebrafish, *dio1* transcripts were higher in liver, and larvae showed a significant increase with exposure to waterborne microcystin-LR, while *dio2* significantly decreased (Yan et al., 2012). In tilapia and crucian carp *Carassius carassius*, the activity of type 1 deiodinase was higher than type 2. Also, the debromination of polybromodiphenyl ethers from flame retardants was species specific and determined by activity of type 1 (Luo et al., 2019). In zebrafish, T3 increased transcript abundance of *igfbp1* (Safian et al., 2016); the testes transcript also associated with TO severity. While there have been several studies on thyroid hormone actions and fish reproductive health (Duarte-Guterman et al., 2014; Tovo-Neto et al., 2018), more research is necessary to understand these interactions.

Estrogen receptors of fishes have been widely studied both in terms of the normal reproductive cycle in fishes, as well as associations with reproductive endocrine disruption. Estrogen receptors *chg*, and *vtg* in the liver are involved in the processes of vitellogenesis and choriogenesis in fishes (Hara et al., 2016) and are sensitive markers of EEDC exposure (Lee et al., 2002). In goldfish *Carassius auratus*, *erβ1* is an estrogen receptor sub-type involved in the regulation of *erα* and vitellogenesis, particularly during spawning (Nelson & Habibi, 2010). The biological and transcriptional associations in female SMB are consistent with spring spawning, including the other positive correlations identified between plasma Vtg and *17βhd*, *erβ1*, *erβ2*, and *chg.*

In male fishes exposed to estrogens, *vtg* and *chg* expression has been correlated with *erα* but not *erβ* subtypes (Marlatt et al., 2008; Sabo-Attwood et al., 2004; Yost et al., 2014). In the male SMB in this study, plasma Vtg was positively correlated with *vtg* and *chg*, as well as *erα*, while it was negatively correlated with *erβ1.* In male zebrafish, *erβ* was downregulated in response to estrogenic exposures (Reyhanian Caspillo et al., 2014; Santos et al., 2014), while being upregulated by exposure to antiandrogens (Filby et al., 2007) and is involved in plasma Vtg induction in males exposed to estrogenic contaminants (Lee Pow et al., 2016). Transcript abundance of *erβ1* was significantly correlated with HSI and plasma Vtg in both sexes. However, in males, it was negatively correlated with HSI and plasma Vtg, while in females it was significantly positively correlated.

Less research has been directed toward androgen receptors and responses to EEDCs, but as with other responses, species differences have been documented (Martyniuk & Denslow, 2012). The abundance of *arα* and *arβ* were negatively correlated with plasma Vtg in male SMB while *arα* was positively correlated with plasma Vtg in females. In Mozambique tilapia sampled from a river impacted by estrogenic contamination, a reduction in *arα* abundance was identified in males with detectable levels of Vtg. Furthermore, this relationship did not exist in male tilapia without plasma Vtg induction from a non-impacted site (Park et al., 2007). The pattern among sites in transcript abundance of *arα* and *arβ* was different, providing further evidence that for species with two AR subtypes, biological processes mediated by a specific subtype may be differentially affected (Bain et al., 2015). Both males and females showed higher abundance of *arβ* at MB compared to the other two sites. Conversely, females from TMC had the highest *arα* abundance, while males from this site had the lowest. *Arα* abundance in males with low TO severity was also significantly downregulated compared to females but not in males with medium or high TO severity. Androgens are key hormones involved in male development and reproduction (Gobinet et al., 2002) and ovarian function (Yu et al., 2018). In this study, the *arα* abundance of males with high and medium TO severity was more similar to females, which may be associated with feminization.

In males with low and medium TO severity, *glk* was significantly downregulated compared to females but was not significantly different between high TO severity males and females. *Glk* in males with high TO severity could indicate an increase in glycogen production associated with increased oocyte severity (Boulekbache, 1981). In order to further validate whether *glk* could be a useful biomarker of TO severity in fish, a better understanding of liver glycogen metabolism during the reproductive cycle in SMB is needed. In a medaka starvation study, a reduction in lipid metabolism (strongly linked to glucose metabolism) caused a female-to-male sex reversal and an increase in the male-related gene, *dmrt1* (Sakae, 2020). It is also suggested that *dmrt1* may be required for male germ cell differentiation and maintenance (Lin et al., 2017). Development of pathway analyses could help tease out the relationship between these transcripts and the role they play in endocrine disruption.

## Conclusion

In summary, common biological indicators of exposure to EEDCs, including TO and plasma vitellogenin, were observed at all three sites despite significant variation in land cover and land use in the watersheds. The variation in multiple transcripts identified in this study and correlations with biological endpoints indicative of exposure to contamination also varied among sites, suggesting differences in environmental stressors. While transcript abundance can change rapidly in response to environmental conditions and associations do not indicate “cause and effect,” it can help to better understand responses to complex mixtures of environmental stressors. It seems likely that there are multiple pathways leading to induction of TO and/or Vtg production in male fishes. The numerous transcripts associated with thyroid activity, insulin regulation, and androgenic responses indicate further research is needed on these potential pathways. Temporal and spatial monitoring of chemicals and biological responses will help to identify associated risk factors and determine if population effects exist.

## Supplementary Information

Below is the link to the electronic supplementary material.

Supplementary file1 (DOCX 20 KB)

## Data Availability

Supporting data can be found at https://doi.org/10.5066/P9RXDWGD.
