# Supplementary material for: Reproductive health and endocrine disruption in smallmouth bass (Micropterus dolomieu) from the Lake Erie drainage, Pennsylvania, USA
Source: Environ Monit Assess. 2021 Dec 4;194(1):3. doi: 10.1007/s10661-021-09654-2 (PMC8643298; doi:10.1007/s10661-021-09654-2)
Supplement: Supplementary file 1 — Supplementary file1 (DOCX 20 KB) [file 10661_2021_9654_MOESM1_ESM.docx]

Supplementary Table 1. De novo transcriptome assembly statistics of male smallmouth bass testes before and after redundant isoform removal with EvidentialGene. Units are defined as base pairs (bp) and n50 (the shortest contig length that needs to be included for covering 50% of the genome).

|  | Original Trinity Assembly | EvidentialGene Results |
| --- | --- | --- |
| Number of Sequences | 132,743 | 50,892 |
| Smallest Sequence Length (bp) | 201 | 201 |
| Largest Sequence Length (bp) | 19,290 | 19,290 |
| Total Number of Bases | 134,609,157 | 69,197,066 |
| Open Reading Frames (#) | 33,163 | 20,007 |
| Mean Open Reading Frame (%) | 51.61 | 53.95 |
| n50 | 2,187 | 2,591 |

Supplementary Table 2. Testes transcript sequences used to create the probes included in the Nanostring nCounter® CodeSet. *Indicates housekeeping transcripts.

| Transcript Name | Transcript Symbol | 100 bp Probe Sequence |
| --- | --- | --- |
| 17-beta hydroxysteroid dehydrogenase | *17βhd* | CATCCTCAACATCTCGTCTGCCAGTGGGATGTACCCTGTTCCTCTCCTCACTGTCTACTCTGCCTCCAAGGCGTTTGTGGACTTCTTCTCCCGAGGACTG |
| 3-beta hydroxysteroid dehydrogenase | *3βhd* | AACTTTTAACTAGAGTCTGGAGGACTGTAGAGGCGACACAAAGTTGAATGTTTTCGAGGGCGACATCAGAGACGGTGATTTCCTGAGAAAACTTGTCGTG |
| 40S ribosomal protein S18* | *40srps18** | GAGAGCTGACTGATGAGGAGGTTGAGCGTGTGGTGACCATCATGCAGAATCCTCGCCAGTACAAAATCCCAGACTGGTTCCTCAACAGGCAGAAGGACGT |
| Aromatase | *cyp19a1a* | GCTGGAGATGGTGATCGCAGCACCAGACACTCTGTCCATCAGCCTCTTCTTCATGCTGCTGCTCCTCAAACAAAATCCAGATGTGGAGCTGAAGCTGCTA |
| Beta-actin* | *βactin** | GTACGTTGCCATCCAGGCTGTGCTGTCCCTGTATGCCTCTGGTCGTACCACTGGTATCGTCATGGACTCCGGTGATGGTGTGACCCACACAGTGCCCATC |
| Cytochrome P450 11B | *cypp45011b* | GAAGCCAGCTGCCATGTGCTCTACGGGGAGCGTATTGGCCTCTTCTCCTCTTCTCCCTCCTTGGAGTCGCAGAAGTTCATCTGGGCCGTGGAGCGGATGT |
| Dehydrogenase reductase SDR family member 11 | *dhrs11* | TTAGCCTTGTCTATCTGCACCCGTGAGGCATACAAATCAATGAAGGAAAGGAATGTGGATGATGGCCACATAATCAATATTAACAGTATGGGTGGGCACC |
| Doublesex and mab-3 related transcription factor 1 | *dmrt1* | GCTGGAGCAGATTGCTTGTTCTCTGTGGAGGCACGATCTCCGACACCTACCAGTACCTCCGCTCCTTCTCTTGTTGTTGCAGGGAGTCGCTCGGCGGCGT |
| Doublesex and mab-3 related transcription factor 2 | *dmrt2* | AGGACGATTTATCAGCGGCATATTCGACCGTCCACCATGCTCGCCAAAAGCATCCTCGAAGGATACTGTCCGGTGCAGTCTGATCCATTCCTGGCGTCCA |
| Doublesex and mab-3 related transcription factor 3 | *dmrt3* | CAGCAGGCCAACGAGAGTCTGGAGAGCCTCATCCCGGAGTCACTCAGAGTGCTGCCCGGTATCGGTATATCCGGAGCCAGCGAGGGGAACCAGGGAGCCC |
| Eukaryotic translation initiation factor 3D* | *etif3d** | ACATGACTCAGTTCAACATGCAGACGCTACCTAAGAGCGCCAAGCAAAAGGAGAGGGATCGTATGCGCCTGCAGAAGAAGTTCCAAAAGCAGTTTGGTGT |
| Follistatin | *fst* | GGTGGGAGTGGATGAGGATATCTCCTCTCTGTTTGTTGAAGATTCGGCCAAGAAGACCCTTGCAAACATTCTTTGGAGAGAAGAAGGTTTGAGTGTGGGG |
| Follistatin 3 | *fst3* | GCATGGGACACCCGGACCTGGAGGTCATGTACCAAGGAGACTGTAAAAAGTCGTGCTCCAATGTGGTGTGCCCAGGTACCCACACCTGTGTGACGGACCA |
| Gonadotropin releasing hormone receptor | *grhr* | CTGTAGCGTGGTGCATGAGTGTCGTGCTGTCAGTCCCTCAGATATTCCTTTTTCACAACGTAACCATCATTCATCCCAAGGACTTCACTCAGTGTACCAC |
| Homeobox protein NOBOX | *nobox* | GTTAGAGCATTTAGAGGCCTTGTTCCAGGAGGACCACTATCCTGACGCAGAGAAGAGGAAAGTCATCGCTGCTTCAGTTGGTGTCACACCTCAAAGAATT |
| Inhibin alpha | *inhα* | ATCTGCCCACTTCTGGTTCTATGCAGGCAAAGGAGCCACATCCAACTCCTCTGCCCAGCTGTTCATTCTCACTTCAGAGCAGAAGCTACTTCAGGCGGCA |
| Inhibin beta b | *inhβb* | TCATCAGCTTTGCGGAGAAAGATGATATGGTGACATCCAAGTCCAGCCTCTTCTTCCAGATCTCCAGTGAGGGGAACCAGAACCTGCATGTGACGCAGGC |
| Insulin like growth factor binding protein 1 | *igfbp1* | CCCTGTCATGTTGAGCTACAGAGAGCCTTGGATAAGATTGCCAAATCCCAGCAGAAATTGGGAGACAAATTAACCAGATTCTACCTCCCTAACTGTGACA |
| Insulin like growth factor binding protein 2A | *igfbp2a* | ATGACCAAAAACCTCATGCCTATAACAATGCTGTCGTACGCGGGCGTCAGCTTGCTAATCCTCTCCGCGTCTCTCGCCGGTGCCTCCCTGGCCGAGATGG |
| Insulin like growth factor binding protein 3 | *igfbp3* | ACCGGGGCAAACACAGGAGAACGCTGGTAATCGGTTAGAAGAGCCTTCTTCCAACGTGACAGCAACGATGACGGTGTTACCTGGTGTGGTGACTGTGAAG |
| Insulin like growth factor binding protein 5 | *igfbp5* | TCTAATTCAAAGCATGAAGGACACTTCCAGGGTCTTGGCTCTCACTCTGCACATTCCCAACTGTGACAAGAAGGGCTTCTTCAAGCGCAAACAGTGTAAG |
| Luteinizing hormone receptor | *lhr* | CCTCATCACTGTCACCAACTCCAAGATTCTGCTGGTGCTCTTCTTCCCAATCAATTCCTGTGCCAATCCCTTCCTCTACGCTATCTTCACCAAGGCTTTC |
| Nanos | *nanos* | ATCTGGCTACTAAATCTACGCTGCCAAACCCTTTGGCTCATCAGCGTCCACCTGATGGTTTGCGGTACGCTTCAGATTCCCTAGGTGCTAATACACCAGA |
| Nuclear receptor subfamily 0 group B member 1 | *nrs0b1* | GCATATCTGAAAGGAGCTGTGCTGTTCAACCCAGATGTGGAAGGTTTGCGCTGTCTTCACTACATCCAGTCTCTGCGTCGGGAGGCGCACCAGGCTTTAA |
| P43 5S RNA-binding protein | *42sp43* | CAAAAAGCGCAGAATGTTTAAACTGCACTTACAGGAGCATGAAGTGGCTGCTAAATTCAAGTGTCTGAAGGACGGATGCGCTGCCACGTTCGACTCCCAT |
| Relaxin receptor 2 | *rr2* | TAAGAGCAGAGAACAACCTGCATGCTGCCTGCATCAAAGTCCTCTGTTGTGCAGACTGCCTCATGGGTGTGTACCTGTTTTTTGTTGGAGTGTTCGATGT |
| Sperm flagellar protein 1 | *spef1* | GCTCCATTTGCGGTCCAGGCAGAGGTGACCCTCAAGACAAACTTCTTCGCCACCAGAGACATGTTGACTCACTTCCTGCCGATCATCAAAGCTGGAGGCC |
| Sperm flagellar protein 2 | *spef2* | CACTATCTTGCAGAAATGAAGAGTATTGACCAACTATCAGAGGTGGTTCGTCACCACATAGAGGCTGGTGCTAAGTTGCAGAATCAGCTGTTATTGGAAT |
| Sperm surface protein SP17 | *sp17* | GCAGCTAATGAAAAAGACAGCATCTCTGTTCCAGACCAAGATATACCTCAGTCTAAGTTAGAGCCCACAGACTTATTATCATACAGAGGGGTTTCAGGTA |
| SRY-box transcription factor 7 | *sox7* | GGGGGCACACATATAGGTCACATCCCTCACATGTCCCAAACTGGAAGTGGCGGTGGACTGATCCCTGGCCATCCGCTGTCTTACTACAGTACCTCATCTT |
| SRY-box transcription factor 9B | *srybox9b* | CTGGCCGATCAATACCCGCATCTGCACAACGCGGAACTCAGCAAAACCCTGGGCAAACTTTGGAGATTGCTCAACGAAGTAGAGAAGCGCCCGTTTGTGG |
| Steroidogenic Acute Regulatory Protein | *star* | AGATGTTGCCTGACATTGGGAAGGTGTTCAAGCTGGAAGTGATGTTGGAGCAGCGTCCTGACAATCTTTACAAAGAGCTGGTGGGAAATATGGAGCAAAT |
| Synaptonemal complex protein 1 | *sycp1* | ACCTTTCAGACGGAGCAACTAAGGAAGGACATCACACAGCATGAAGTAAAGTACAAAGAACTATTATCCAACTTTAACGAGCTCCAGTCTGAGAAGGCAG |
| Uncharacterized protein 1 | *up1* | ATGAACCCAGCTACGCCTCCAGTGAGCAGATTACCAGCAGTACAGGCTCTCTCCAAGCCTCTTGGAACAGCAGGAACTCTGCCCAGGGAGCTCGTAACCT |
| Uncharacterized protein 2 | *up2* | CTGGACATCAACTACACACTGACTGTTCTGCCTGAAAACGAACTGTATTACCACCTGGCATCTATTGAGGCATTGTTCACAGATGTCTATCCTCCATCCT |
| Vitellogenin | *vtg* | AAATACTTCAAGTCGTATGACAATGGTGACTCTCAGCTGGACTCCTCAGAGCTGCTCAAATTCATCCAGCAAAATGACTCGGTTGTGGAGTTGCAGTCTT |
| Vitellogenin C | *vtgc* | TTCCAAGAAAGCCACCCAGCGAGTACGTGTGTCCTCTGATTCAGCCTCAAGCGTCAGAGAGAGATCTAATCACAGCCATCATGACAATATAATGGAAAGC |
| Wnt family member 5B | *wnt5b* | GGGCGGTTGCGGCGACAACGTACATTATGGCTACCGATTTGCCCGGGAGTTTGTGGACGCCAGGGAGAGAGAGAAGAATTACCCGCGTGGTTCTGCTGAG |
| Zona pellucida 3 | *zp3* | TGGGAGTCATCTGTTGTACACCAATGATCTGACCTACATTTCCTCTCCCGATTCTAACATTCTCCCGTCCACTCACCTAGTTGTCTGTGAGTATGAGAGG |
| Zona pellucida 3iX1 | *zp3ix1* | CAAGAGTTATGCTGTGGATGGCATTTCCCTGCATCCCACCTGGGTTCCTTTTGTCAAAACAGCCTCAGCAGAGGATCAGCTGGTCTTCAATTTGCGAACC |
| Zona pellucida 4 | *zp4* | GACACTGAAGCTCTACAAGTCGTATTTGGATCTCTGTACCGGGATGATGTTCTGATCAAGCCCAGCAGAGTCGTCAGTATTCTTGCCGCTGCTTGTATGC |
| Zona pellucida AX | *zpax* | GGTTACAGTCTACTCCCTCCCTCTGATCTTTGGGCTCATTATCCTACCTGAACAAATGCAGTTCACTCACTCAGTGGATTTGCAGGCGTCTCTGCAGGAT |
| Zygote arrest 1-like | *zar1* | GGGTCCAACTTTCAGTTCCTAGAGCAGAGGTATGGCTTTTTCCACTGCAAAAAGTGTAACATCCGGTGGGAGAGTGCTTATGTGTGGTGCATCTCTGGAA |

Supplementary Table 3. Liver transcript sequences used to create the probes included in the Nanostring nCounter® CodeSet. *Indicates housekeeping transcripts.

| Transcript Name | Transcript Symbol | 100 bp Probe Sequence |
| --- | --- | --- |
| 17-beta Hydroxysteroid Dehydrogenase | *17βhd* | CATCCTCAACATCTCGTCTGCCAGTGGGATGTACCCTGTTCCTCTCCTCACTGTCTACTCTGCCTCCAAGGCGTTTGTGGACTTCTTCTCCCGAGGACTG |
| 40S Ribosomal Protein S12* | *40SrpS12** | CTCCCTGATCCACGATGGCCTTGCCCGTGGTATCCGTGAGGCCACAAAGGCCCTGGACAAGCGTCAGGCTCATCTCTGCGCTCTGGCTGCCAACTGCGAT |
| Androgen Receptor Alpha | *arα* | GACTGCACCATCGACAAACTGAGGCGGAAAAACTGCGCCTCGTGCCGTTTAAAGAGATGCTTCATGTCGGGAATGAGCCTTAAAGGTCGCAGGCTAAAGG |
| Androgen Receptor Beta | *arβ* | TCGATAAGCTAAGAAGAAAGAACTGTCCGTCGTGTCGGCTGAAAAAGTGTTTTGAAGCTGGAATGA CTCTCGGAGCACGCAAACTGAAGAAGATTGGACA |
| Aryl Hydrocarbon Receptor | *ahr* | CGCCGCCTCAGCTCGCCCTGTTCGCCATCGCCACACCCCTCCAGCCTCCAGCCATCCTTGAAATCAGGACGAGGAACATGATTTTCAGGACCAAACACAA |
| Choriogenin | *chg* | GAAAGCAAAAATGTGAGCAGCCTTCCTCTTGACCCTCTATGGGTCCCATTTTCTGCGGTTAAGGTGGCTGAGGAATTCTTATACTTCACCCTGAAACTCA |
| CYP1A | *cyp1*α | CAGCTCAACACTGTTATGAAGGCTGAAGGCAGTTTCGACCCATTCCGCTACATCGTTGTCTCTGTTGCTAATGTGATCTGTGCCATGTGCTTTGGCCGAC |
| CYP3A | *cyp3*α | TTCGGCACTATGCTGGCATATAAAAAGGGATTCATGCACTTTGATTCGGAGTGCTTCAAGAAATATGGGAAAACATGGGGCATTTTTGATGGCCGTCAGC |
| Elongation Factor 1A* | *ef1α** | ATGATGACCTGGGCGTTGAAGTTGTCAGCTCCCTTGGGTGGGTCGTTCTTGCTGTCGCCAGCCACGTATCCACGACGGATTTCCTTGACGGACACGTTCT |
| Estrogen Receptor A | *erα* | CTCTCCCACCATCAGGCACATGAGCGAACAAAGGTAATGGAGCATCTCTACAGCATGAAGTGCAAGAACAAAGTGCCTCTGTACGACCTTCTGCTGGAGA |
| Estrogen Receptor Beta 1 | *erβ1* | GCCTGAGCAGAGAAGAGGGGAGCTGTGTCCAGGGCTTCGCAGAGATCTTTGATATGCTGATAGCTGCCACGTCCAGGGTGAGAGAGCTCAAGCTCCAGAG |
| Estrogen Receptors Beta 2 | *erβ2* | CTGCAGCTCCATCCTCGGTTGTAACAAGCCGCTGGGGAAGAGATTAGAGGAAGGAGTGGAAGGCGTGAACTCCTCCTTGTGTTCTTCTGCGGTAGGGAAA |
| Eukaryotic Translation Initiation Factor 3D* | *etif3d** | ACATGACTCAGTTCAACATGCAGACGCTACCTAAGAGCGCCAAGCAAAAGGAGAGGGATCGTATGCGCCTGCAGAAGAAGTTCCAAAAGCAGTTTGGTGT |
| Glucokinase | *glk* | TACTCTATTCCTGAAGATGCCATGACAGGCACTGCCGAAATGCTATTTGATTACATAGCAGAGTGCATGTCAGACTTCTTGGACAAACATCATCTCAAGC |
| Insulin-like Growth Factor 1 | *igf1* | CGGAGACCCGTGGGGATGTCTAGCGCTCTCTCCTTTCAGTGGCATTTATGTGATGTCTTCAAGAGTGCGATGTGCTGTATCTCCTGTAGCCACACCCTCT |
| Metallothionein | *mt* | CAGCGCTCTACTGCCCTTGTGATGGCGCCTGTGTGAACAACTAATGACTAACTGCACATGTCTACATAAAATGTATTTTGTACTCGTCTCAGCGTTGCAG |
| Ribosomal Protein L8* | *rpl8** | TTCTCCTCTGCAAACAGAGCTGTTGTTGGTGTGGTAGCTGGAGGTGGTCGTATTGACAAGCCCATCCTGAAGGCTGGTCGTGCCTACCATAAGTACAAGG |
| Thyroid Hormone Receptor Beta | *thrβ* | TGCATTACCTGTGAAGGTTGCAAGGGTTTCTTCAGGCGGACGATCCAGAAGAATCTCAACCCTACCTACGCCTGTAAGTATGAGGCGAAATGCGTCATCG |
| Type I Deiodinase | *dio1* | GCTGTGAAATTGTGAGTTCACCACCAAGGAGCAAGCATGTTTTTGCAAAGACTGATGGTCTATTTATCGACAGTATGCCTGTTTTGCTATATGATAGGAC |
| Type II Deiodinase | *dio2* | ACTGTCTGGAGCAGTCCACAGTGCAGAATACAAACTATGAGTAGGACGACTGAGACCGACGATGCAGCGTTGTGCGTAATGAAGGGTAGTCTGGAATAAA |
| Vitellogenin | *vtg* | CATTGGTAATCATGTTGCGCTGAGGTTCATCAAGGAGCACTTCCTCGCTGGTGAGCTATCTTTTGCTGAAACTGCTCAAGCCCTGCTGGCATCTGTTCAC |
